# Cross-reactive memory T cells associate with protection against SARS-CoV-2 infection in COVID-19 contacts

**DOI:** 10.1038/s41467-021-27674-x

**Published:** 2022-01-10

**Authors:** Rhia Kundu, Janakan Sam Narean, Lulu Wang, Joseph Fenn, Timesh Pillay, Nieves Derqui Fernandez, Emily Conibear, Aleksandra Koycheva, Megan Davies, Mica Tolosa-Wright, Seran Hakki, Robert Varro, Eimear McDermott, Sarah Hammett, Jessica Cutajar, Ryan S. Thwaites, Eleanor Parker, Carolina Rosadas, Myra McClure, Richard Tedder, Graham P. Taylor, Jake Dunning, Ajit Lalvani

**Affiliations:** 1 NIHR HPRU in Respiratory Infections, Imperial College London, London, England; 2 National Heart and Lung Institute, Imperial College London, London, England; 3 Section of Virology, Department of Infectious Disease, Imperial College London, London, England; 4 National Infection Service, Public Health England, London, England; 5 NIHR HPRU in Emerging and Zoonotic Infections, London, England

**Keywords:** Antimicrobial responses, Viral infection, SARS-CoV-2, Immunological memory

## Abstract

Cross-reactive immune responses to SARS-CoV-2 have been observed in pre-pandemic cohorts and proposed to contribute to host protection. Here we assess 52 COVID-19 household contacts to capture immune responses at the earliest timepoints after SARS-CoV-2 exposure. Using a dual cytokine FLISpot assay on peripheral blood mononuclear cells, we enumerate the frequency of T cells specific for spike, nucleocapsid, membrane, envelope and ORF1 SARS-CoV-2 epitopes that cross-react with human endemic coronaviruses. We observe higher frequencies of cross-reactive (p = 0.0139), and nucleocapsid-specific (p = 0.0355) IL-2-secreting memory T cells in contacts who remained PCR-negative despite exposure (n = 26), when compared with those who convert to PCR-positive (n = 26); no significant difference in the frequency of responses to spike is observed, hinting at a limited protective function of spike-cross-reactive T cells. Our results are thus consistent with pre-existing non-spike cross-reactive memory T cells protecting SARS-CoV-2-naïve contacts from infection, thereby supporting the inclusion of non-spike antigens in second-generation vaccines.

## Abstract (web summary)

While cross-reactive immunity between human coronavirus and SARS-CoV-2 may contribute to host protection, validating evidences are still scarce. Here the authors assess a cohort of 52 donors with immediate-early contact with SARS-CoV-2 to correlate higher frequency of cross-reactive T cells with lower infection rate.

## Introduction

Despite mass deployment of effective vaccines against SARS-CoV-2, correlates of protection against infection remain unknown. Exposure to SARS-CoV-2 does not universally result in infection and pre-existing T cells, primed by endemic human coronaviruses (huCoVs), might mediate protection in SARS-CoV-2-naive persons. Studies to date have described the prevalence of SARS-CoV-2 cross-reactive T cells in naive healthy controls^1–4^ and in hospitalised COVID-19 patients^5,6^. However, no study yet describes an association of cross-reactive T cells with outcome after SARS-CoV-2 exposure.

Here we assess contacts of newly diagnosed COVID-19 cases to capture the earliest time-points after SARS-CoV-2 exposure. We quantify T cells specific for in silico-predicted and biologically confirmed pools of cross-reactive epitopes from 5 SARS-CoV-2 proteins, alongside protein-spanning peptide pools, using a highly sensitive dual cytokine fluorescence-linked immunospot (FLISpot) assay to detect both IFN-γ and interleukin-2 (IL-2). The frequency of baseline cross-reactive T cells is correlated with the infection outcome following SARS-CoV-2 exposure, and we observe significantly higher frequencies of cross-reactive memory T cell responses in PCR-negative contacts. The association of circulating SARS-CoV-2-specific T cells at exposure with lack of infection is the first evidence of a protective role for cross-reactive T cells in COVID-19, and establish the potential for second-generation T cell-inducing SARS-CoV-2 vaccines that could circumvent spike-antibody immune escape variants.

## Results

### SARS-CoV-2 proteins contain heterologous and homologous epitopes predicted to cross-react with huCoVs

**Fig. 1:**
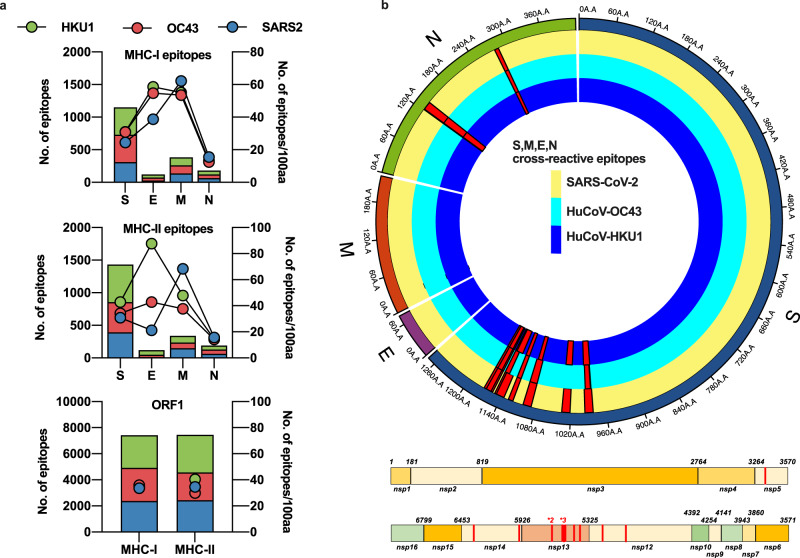
In silico prediction of putative epitopes in human endemic beta-coronaviruses and SARS-CoV-2. Available sequences for S, M, E, N and ORF1 from SARS-CoV-2, huCoV-OC43 and huCoV-HKU1 were assessed for potential HLA-binding motifs by SYFPEITHI and IEDB as described in the Methods. The number of predicted epitopes is presented in the stacked bar charts and the immunogenicity for each protein of each virus is depicted as points on the right *Y*-axis. Green bars and circles depict huCoV-HKU1 (HKU1), red huCoV-OC43 (OC43) and blue SARS-CoV-2 (SARS2) (**a**). The predicted cross-reactive epitopes and their prevalence within each virus for all sequences available are mapped as red histograms in a linear plot for ORF1 and a radial plot for S, M, E and N. All mapped cross-reactive epitopes within ORF1 were present across SARS-CoV-2, HKU1 and OC43. Within the radial plot, the yellow track represents SARS-CoV-2 sequence, dark blue HKU1 and light blue OC43, with red histograms representing the prevalence of the putative epitope across the viruses (**b**).

We compared the frequency of early cross-reactive T cells in SARS-CoV-2 PCR-positive and PCR-negative COVID-19 contacts identified through rapid contract tracing, using a cross-reactive peptide pool defined by a novel bioinformatic approach. Previous studies have applied whole viral proteome-spanning peptide pools to interrogate pre-pandemic cohorts for pre-existing cross-reactivity. Approaches using restricted numbers of epitopes^3,4,7^ have demonstrated greater prevalence of cross-reactive responses than studies employing large pools^1,2,8^. We defined a set of cross-reactive epitopes identified by cross-referencing predicted MHC-binding motifs within alignments of huCoVs and SARS-CoV-2 protein sequences, in addition to direct epitope prediction from regions of high shared homology, as has been applied more conventionally^9^. This required sufficient sequence homology to generate alignments, which was possible for spike (S), nucleocapsid (N), membrane (M), envelope (E) and ORF1 proteins between SARS-CoV-2 and the beta-coronaviruses OC-43 and HKU1. The number of epitopes identified was largely dependent on the size of the protein; however proteins E and M had a greater number of epitopes when normalised for gene length for all three viruses (Fig. 1a). ORF1 had the highest homology between the human beta-coronaviruses and SARS-CoV-2, where 12 fully conserved homologous MHC-I epitopes were identified, along with one conserved epitope in nucleocapsid. We identified 17 examples of a heterologous epitopes that would bind the same HLA allele within the same position within the alignment within 15 regions of S and N (Fig. 1b and Supplementary Table 1) between HKU-1 and OC43 and SARS-CoV-2. These epitopes were predicted to bind a broad set of HLA alleles (Supplementary Table 1), including ones common in the European population (e.g., HLA-A*02:01 present in >50% of the European population, HLA-B*40 in >30% and HLA-DRB1*01 in >30%^10^). Nelde et al.^3^ identified SARS-CoV-2-specific IFN-γ -secreting T cells in pre-pandemic cohorts through extended culture; therefore, we included 14 of the most prevalent of these in vitro-confirmed cross-reactive epitopes alongside our in silico-predicted epitopes.

### Higher baseline frequencies of cross-reactive IL-2 secreting T cells associate with protection from infection in COVID-19 contacts

**Fig. 2:**
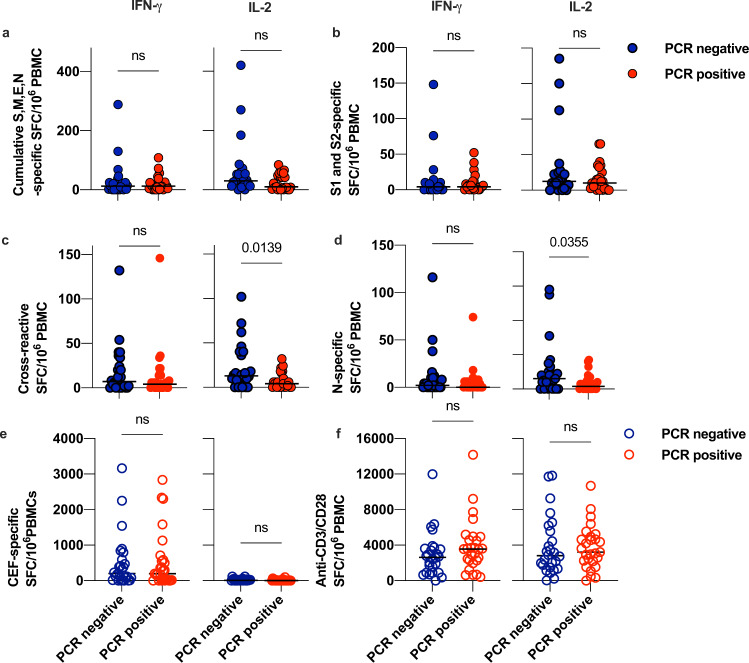
Dual cytokine FLISpot responses to SARS-CoV protein-spanning peptide pools and the cross-reactive pool in PCR-positive and PCR-negative COVID-19 contacts. PBMCs sampled from COVID-19 contacts (*n* = 52) at the baseline visit were rested overnight at high density prior to stimulation with 1 µg/ml/peptide pools, 1 µg/ml CMV-EBV-Flu (CEF) positive control peptide pool or 1 µg/ml anti-CD3 and 1 µg/ml anti-CD28 antibody as indicated and cultured for 20 h in a FLISpot assay to detect IL-2- (left-hand panels) and IFNγ (right-hand panels) secreting T cells. Data are DMSO-subtracted spot forming cells per 1 × 10^6^ PBMC. Blue circles represent PCR-negative contacts and red circles represent PCR-positive contacts. Filled circles represent test data from SARS-CoV-2 stimuli and the outline circles represent values from positive control stimuli. **a** Depicts cumulative frequency of pan-S,M,E and N-specific T cells using protein-spanning 15-mer peptide pools to represent the entire protein, rather than predicted epitopes, as a stimuli. **b** Depicts the frequency of pan-S-specific T cells, using protein-spanning 15-mer peptide pools to represent the entire protein, rather than predicted epitopes, as stimuli. **c** Depicts the frequency of cross-reactive T cells, using peptides derived from the putative MHC-I and MHC-II epitopes defined in our bioinformatic analysis and Nelde et al, as listed in Supplementary Table 1. **d** Depicts the frequency of pan-N-specific T cells using protein-spanning 15-mer peptide pools to represent the entire protein, rather than predicted epitopes, as a stimuli. **e** Depicts the frequency of T cells specific to an antigen-specific positive control peptide pool comprising of well-characterised epitopes from influenza, cytomegalovirus and Epstein–Barr Virus. **f** Depicts the frequency of IL-2 and IFNγ secreting T cells activated in response to a polyclonal stimulus of anti-CD3 and anti-CD28 soluble antibody. Data are from *n* = 26 PCR-positive and *n* = 26 PCR-negative contacts and *p*-values are from a two-sided Welch’s *T*-test.

PBMCs sampled from the 52 confirmed exposed contacts within the INSTINCT study at 1–6 days post-index symptom-onset were assayed for IFN-γ and IL-2-secreting T cell responses to the SARS-CoV-2 S, M, E and N peptide pools, as well as the cross-reactive pool containing the 28 epitopes we identified and the 14 epitopes from Nelde et al., (Supplementary Table 1). We were able to detect responses to SARS-CoV-2 peptide pools in both PCR-positive (*n* = 26) and PCR-negative (*n* = 26) contacts (Fig. 2a–d). We observed no significant difference in the cumulative frequency of IFN-γ or IL-2-secreting T cells in response to the SARS-CoV-2 S, M, E and N-spanning pools between individuals that were infected and those that remained uninfected (Fig. 2a, Welch’s *T*-test, *p* = 0.4206). The S-protein of SARS-CoV-2 is one of the most commonly reported sources of cross-reactive responses in pre-pandemic cohorts^1^. Whilst we did observe S-specific IFN-γ and IL-2 secreting T cells in exposed contacts at the baseline visit, we saw no significant difference in frequencies of these responses between PCR-positive and PCR-negative contacts (Fig. 2b). In contrast, there were significantly higher frequencies of IL-2-secreting cross-reactive T cells in exposed contacts that remained PCR-negative (Fig. 2c, Welch’s *T*-test, *p* = 0.0139), but no difference in the frequency of IFN-γ -secreting cells. nor in the frequency of dual positive IL-2/IFN-γ -secreting T cells specific for the cross-reactive pool. The cross-reactive IL-2 secreting T cells associated with absence of infection did not co-produce IFN-γ, suggestive of a pre-existing antigen-specific memory T cell population. The frequency of IL-2 secreting cross-reactive T cells had an odds ratio (OR) of 1.06 (95% CI: 1.011–1.12, *p* = 0.0295) for a PCR-negative result in an unadjusted binary logistic model. The PCR-negative contacts also had higher frequencies of pan-N-specific IL-2-secreting cells (Fig. 2d, Welch’s *T*-test, *p* = 0.0355), however we observed no significant protective effect in a binary logistic regression model. There was no difference in the relationship score of the contact with their index case (Table 1, Mann–Whitney *U* test, *p* = 0.5004), or days since index symptom-onset for the baseline visit (Table 1, Mann–Whitney *U* test, *p* = 0.2935), for PCR-positive versus PCR-negative contacts, suggesting similar SARS-CoV-2 exposure between the groups.

**Table 1:** Demographics for the whole cohort, PCR-positive and PCR-negative contacts of confirmed COVID-19 cases.

	Whole cohort	PCR-negatives	PCR-positives	*p*-value (Mann–Whitney *U*, two-sided)
Age	(*n* = 52)	(*n* = 26)	(*n* = 26)	
Median (IQR)	33 (23–49.25)	32 (21.25–48)	33 (27.75–51)	0.4156
Days since symptom onset in index (V1)	(*n* = 52)	(*n* = 26)	(*n* = 26)	
Median (IQR)	5 (4–5)	4.5 (3–5)	4 (3–5)	0.2935
Days since symptom onset in contact (V1)				
Median (IQR)	2.5 (1.75–3.25)	NA	2.5 (1.75–3.25)	NA
Ethnicity	(*n* = 52)	(*n* = 26)	(*n* = 26)	
White (British or other)	43 (82.7%	20 (76.9%)	23 (88.5%)	
Hispanic	3 (5.7%)	3 (11.5.69%)	0 (0%)	
Not Stated	5 (9.6%)	3 (11.5%)	2 (7.7%)	
Afghan	1 (1.9%)	0 (0%)	1 (3.8%)	
Male:Female ratio	(*n* = 52)	(*n* = 26)	(*n* = 26)	
Median (IQR)	1.08:1	1.16:1	1:1	>0.999*
Relationship score	(*n* = 46)	(*n* = 23)	(*n* = 23)	
Median (IQR)	80 (60–100)	80 (80–100)	60 (60–100)	0.5004
100	16	7	9	
80	15	11	4	
60	12	5	7	
40	1	0	1	
20	3	1	2	
10	0	0	0	
Lymphocyte count	(*n* = 32)	(*n* = 12)	(*n* = 20)	
Median (IQR)	1.6 (1.3–2.0)	1.3 (1.025–1.6)	1.7 (1.425–2.4)	0.0039

*P-value from Fisher’s Exact test.

### Cross-reactive T cells are induced by SARS-CoV-2 infection

**Fig. 3:**
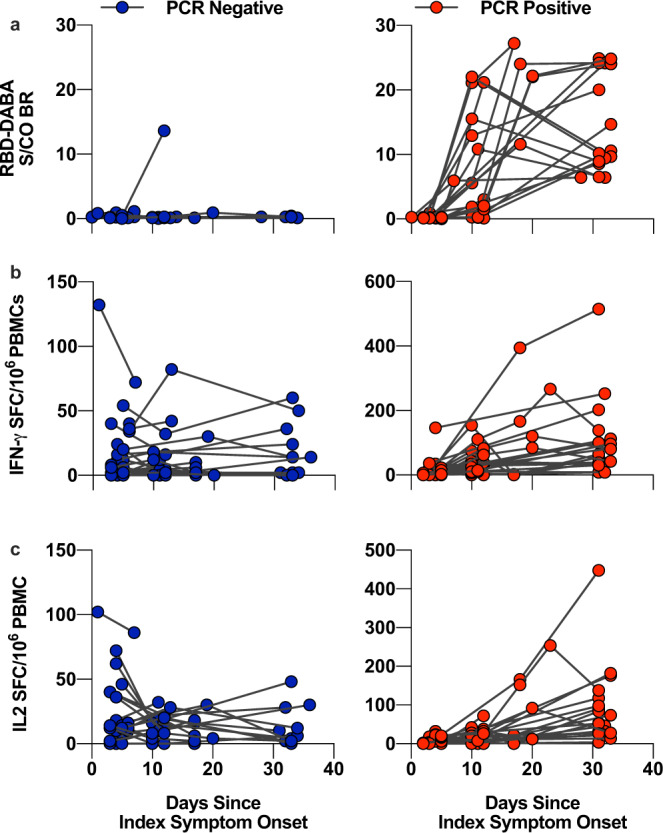
Dynamics of cross-reactive T cells and RBD-specific antibody in PCR-positive and negative contacts. Serum sampled from COVID-19 contacts at the baseline, D7 and D28 visit were assayed for RBD-specific antibody, represented as sample/control ratios (**a**). PBMCs from these visits were rested overnight at high density prior to stimulation with 1 µg/ml cross-reactive peptide pool cultured for 20 h in a FLISpot assay to detect IL-2- (**b**) and IFNγ (**c**) secreting T cells. Serum from these visits were assayed for RBD-specific antibody, represented as sample/control ratios (**c**). Left-hand panels and blue circles represent PCR-negative contacts whilst right-hand panels and red circle represent PCR-positive contacts.

Although the PCR-positive contacts had mild, ambulatory COVID-19, they did have significantly lower lymphocyte counts than the PCR-negative contacts (Table 1). Despite this, frequencies of IFN-γ and IL-2 secreting T cells (Fig. 2e) responding to antigen-specific and polyclonal positive controls (Fig. 2e, f) in infected contacts were not reduced compared to those who remained uninfected. Following exposure, 91% of PCR-positive contacts with follow up samples (*n* = 22) developed RBD-specific antibodies (Fig. 3a), thus serologically confirming infection. Furthermore, the same individuals also showed strong induction of both IFNγ and IL-2-secreting functional subsets of T cells specific for the highly conserved epitopes, confirming the cross-reactivity of these epitopes with SARS-CoV-2 (Fig. 3b, c). For a subset of the non-homologous cross-reactive epitopes from S and N proteins, we had huCoV-specific corresponding peptides (Supplementary Table 2). We observed equivalent induction of SARS-CoV-2-specific S/N reactivity as for huCoV-specific S/N for both IFNγ (Supplementary Fig. 1, Pearson’s *r* = 0.9052, *p* = 0.0001) and IL-2 (Pearson’s *r* = 0.8249, *p* = 0.0001) secreting T cells. This could be due to heterologous recognition by the same set of T cell receptors.

Interestingly, following SARS-CoV-2 exposure, we observed dynamic changes in the frequencies of cross-reactive IL-2 secreting T cells in PCR-negative contacts with a baseline response (Fig. 3b, c, Supplementary Fig. 2, *p* = 0.0039 in a maximum-likelihood method mixed-effects analysis). No significant changes were observed in frequencies of T cells specific to control non-SARS-CoV-2 viral peptides from CMV, EBV and influenza (Supplementary Fig. 2). The peripheral depletion of cross-reactive T cells in PCR-negative contacts is notable and implies an active response to the temporally related SARS-COV-2 exposure, potentially through migration from blood to the respiratory mucosa.

Previous work has suggested a higher prevalence of endemic coronaviruses infections in younger individuals^11^ and reduced prevalence of huCoV-specific T cells in older adults^12^. Neither the frequency of IL-2-secreting cross-reactive SARS-CoV-2-specific T cells (Spearman’s *r* = −0.1744, *p* = 0.2162), nor the PCR status of contacts (Mann–Whitney *U* test *p* = 0.5863) were associated with age; however, the age range of the cohort was not wide, with very few children (Table 1, median 33.04 years of age, IQR 25.30–50.04).

## Discussion

Our study suggests that the initial frequency of IL-2-secreting cross-reactive T cells is associated with protection from infection in COVID-19 contacts. The prevalence of cross-reactive T cells, primed by exposure to huCoVs, has often been suggested as a factor influencing outcome after SARS-CoV-2 exposure. To date, however, evidence of whether these T cells could provide such a protective effect has been lacking. Some reports have suggested that SARS-CoV-2 infection may induce T cells without seroconversion^13^; however, this is unlikely to be the source of the cross-reactive T cells we observe in PCR-negative individuals. The rapid sampling of contacts within 1–6 days of symptom-onset in index cases, with de novo induction of IFN-γ and IL-2-secreting SARS-CoV-2-specific T cells occurring after 10 days in PCR-positive contacts (Fig. 3b, c), strongly suggest that the SARS-CoV-2-reactive T cells we quantified were pre-existing. Furthermore, the IL-2-only secretion status is indicative of a pre-existing memory T cell phenotype rather than de novo expanded T cells in response to recent antigen exposure. It is conceivable that reports of T cell responses without seroconversion^13,14^ could be related to rapid expansion of pre-existing cross-reactive T cells promptly controlling infection and thereby abrogating subsequent induction of de novo SARS-CoV-2-specific antibody responses. A major epitope within our predicted pool has been demonstrated to be immunodominant in convalescent COVID-19 patients^15,16^, concordant with the idea that pre-existing T cells may be preferentially expanded.

We observed de novo induction of both IFN-γ and IL-2-secreting T cells in PCR-positive individuals, confirming SARS-CoV-2 reactivity of the peptides used within our cross-reactive pool. The higher prevalence of huCoV-seropositivity in PCR-negative contacts (*p* = 0.029 Fisher’s Exact test, Supplementary Fig. 3) together with the association of seropositivity with baseline frequencies of cross-reactive IL-2-secreting T cells (*p* = 0.0432 Mann–Whitney *U*) in PCR-negative contacts supports the notion that these pre-existing T cells were induced by prior huCoV exposure SARS-CoV-2 cross-reactive antibodies have been described for the S2-region of S protein from beta-huCoVs^17^, with boosting observed in SARS-CoV-2 infection^18^. Within our study, we surmise, as have others^19^ that huCoV-antibodies are a marker of prior huCoV exposure while the cross-reactive memory T cells mediate protection; however, it is also possible that the antibodies contribute to or mediate protection themselves.

Wyllie^20^ et al. have demonstrated that IFN-γ -secreting T cells specific for SARS-CoV-2-exclusive epitopes induced by prior symptomatic SARS-CoV-2 infection are associated with protection from reinfection in a prospective study of healthcare workers with low anti-S antibodies. This complements our novel finding that IL-2-secreting T cells responding to exclusively cross-reactive epitopes, indicative of memory T cells from previous huCoV infection, may protect against infection in SARS-CoV-2-naive seronegative individuals. Both their and our observations are surprising, as the prevailing hypothesis has been that T cells, be they SARS-Cov-2-induced homologous or huCoV-induced heterologous, would limit viral load or symptom burden^21^, as has been observed for influenza^22^. It was not possible for our study to correlate baseline cross-reactive T cells with symptom severity or peak viral load as such T cells were completely absent in all the PCR-positive contacts, so clear-cut was the protective association observed.

It is perhaps unsurprising that the signal we detected was modest because we measured ex vivo responses of physiological relevance based on detection of IL-2 secretion by T cells within 20-h of antigen contact without co-stimulation. Our background IL-2 response to DMSO negative control was low (median = 4.5 and IQR 2–7.8) and the use of a stringently selected set of high-purity cross-reactive peptides in the absence of exogenous co-stimulation or antigen-driven expansion during extended culture mean the cross-reactive T cells we detected are unlikely to be non-specific artefacts. Furthermore, these cross-reactive IL-2-secreting T cells can be stimulated with cognate antigen in vitro to proliferate and expand into IFN-γ -secreting T cells (Supplementary Fig. 4) and therefore likely represent memory T cell responses^23^. In contrast, IFN-γ -secreting T cells specific for cross-reactive epitopes could not be grown from individuals who lacked IL-2-secreting T cells. This is consistent with the phenotype of huCoV memory T cells described previously^1^. Other studies employing ex vivo IFN-γ ELISpot have not consistently detected pre-existing responses in uninfected or pre-pandemic cohorts^4,24^ unless enumerating ORF1 responses, which appear to be amongst the most predominant sources of cross-reactive T cells in SARS-CoV-2-naive-individuals^4^. A systematic appraisal of the sensitivity of different assay to detect cross-reactive T cells in pre-pandemic cohorts revealed a high-prevalence of spike-directed responses with proliferation assay, but not IFNγ ELISpot^25^. The wider success of such proliferation assays is indicative of a memory population and as such an assay capturing IL-2-responses is more likely to identify an ex vivo signal.

Ours is the first study to detect ex vivo IL-2 responses, that likely correlate with central memory T cell responses^23,26,27^, specific for cross-reactive epitopes in contacts of confirmed COVID-19 cases, early after exposure. We had insufficient cell numbers to perform flow-cytometry or immunomagnetic depletion to confirm the cell surface marker phenotype of the cross-reactive T cells. We could not delineate fine epitope specificity; however, these T cells are likely ORF1- and N-specific. Apart from spike, ORF1 and N were the major contributors to the cross-reactive peptide pool (Supplementary Table 1) and frequencies of IL-2-secreting T cells responding to N-spanning peptide pools were higher in uninfected contacts, with no difference observed for pan-S-specific T cells (Fig. 2c). In accordance with other studies, we were able to detect robust responses to SARS-CoV-2 S protein, but these were not associated with protection from infection. Bacher^6^ et al., observed low affinity huCoV spike-specific CD4 T cells had low functional avidity against SARS-CoV-2 in COVID-19 patients, consistent with our observation that initial responses to S-protein were not associated with protection. As such, the higher frequency of T cells specific for the cross-reactive pool in SARS-CoV-2 exposed PCR-negative contacts implies ORF1 and N-, not S-, reactive T cells play a protective role. This finding is consistent with the higher prevalence of cross-reactive ORF1 and N-specific immune responses than S-specific immune responses in some pre-pandemic cohorts^4,28^. A compelling study in healthcare workers has shown higher frequencies of IFNγ-producing ORF-1 reactive T cells to be associated with a lack of infection as determined by the absence of seroconversion during the first wave of the pandemic in the UK^29^. This higher frequency was presumed to be an expansion of pre-existing T cells from prior huCoV exposure, consistent with our finding that the cross-reactive memory T cells were associated with protection from infection.

It should be noted that 19/26 exposed uninfected contacts had no appreciable IL-2 response (i.e. >22 SFC/10^6^ PBMC) to our cross-reactive pool. This is consistent with the inevitability that the mechanisms behind the phenomenon of exposed uninfected individuals are polyfactorial. Shedding characteristics of index cases and the behavioural choices of the contact will contribute to the level of exposure, whilst genetic polymorphisms and other demographic factors will influence innate immune responses that could modulate susceptibility to infection^30^. The relative importance of the various host-intrinsic mechanisms may differ with varying levels of exposure and SARS-CoV-2 inoculum. As such, the relative importance of cross-reactive T cells as one of these mechanisms may vary according to infectious inoculum, environmental and genetic factors. Our study is small, with participants of predominantly White-European ethnicity (88%, Table 1), thus limiting our ability to model additional demographic factors in our study. However, the targeted study design and use of highly specific cross-reactive epitopes has provided a clean signal to delineate a significant protective association of cross-reactive T cells in this population.

Our in silico to ex vivo approach in defining cross-reactive epitopes has rarely been applied to SARS-CoV-2. Whilst we were able to detect a significant difference in frequencies of cross-reactive T cells between PCR-positive and -negative individuals in our study, it is quite possible that our approach is missing some cross-reactive epitopes. HuCoVs were underrepresented in available sequencing depositories at the beginning of the COVID-19 pandemic, likely limiting our ability to robustly represent all potential, or more contemporary, epitopes derived from past huCoV exposure. Reliance on MHC-binding prediction algorithms rather than biological data may have skewed the epitopes identified towards MHC-alleles that are better characterised.

The emergence of novel variants with potential to escape naturally acquired or vaccine-induced humoral immunity, along with the recent elucidation of immune-mediated antigenic drift in huCoVs^31^ brings the long-term utility of spike-only based vaccines into question. We demonstrate the importance of non-spike targets, in particular ORF1 and nucleocapsid, for T cell-mediated protection in the absence of neutralising antibodies, consistent with the wide spectrum of antigen-specific T cells induced by SARS-CoV-2 infection^5,13,24,32^ and cross-reactive T cells in pre-pandemic cohorts^4^. In light of this, inclusion of these targets alongside the major antibody target of S-protein could be critical in maintaining the benefit of vaccination in the case of vaccine-strain mismatch, as could occur with the emergence of novel variants^33^. Our study complements the small but growing body of evidence that T cells may protect against SARS-CoV-2 infection and supports the potential utility of second-generation vaccines targeting core proteins^22,29,34^.

## Methods

### Study design and participants

Samples and participant data were obtained and biobanked under the INSTINCT study (IRAS: 282820, REC reference 20/NW/0231 approval granted by North West Greater Manchester East Research Ethics Committee) within the National Institute of Health Research (NIHR) Health Protection Research Unit (HPRU) in Respiratory Infections at Imperial College London. SARS-CoV-2 positive individuals were identified through Public Health England (PHE) and the National Test and Trace (NTAT) programme in the United Kingdom. Research nurses obtained informed consent for home visits and sampling, having obtained consent to contact potential participants about the study via PHE. Participants received no compensation for participation. Serum was obtained from clotted blood samples at all times points and peripheral blood mononuclear cells (PBMCs) were separated by density centrifugation of heparinised blood at the baseline visit. PBMCs were cryopreserved prior to fluorescence-linked immunospot (FLISpot) analysis.

Participants provided nasopharyngeal swabs at the baseline, day 4 and day 7 visit and RT-PCR performed to detect SARS-CoV-2 E gene (10 μl RNA in a 20 μl reaction, forward primer 5'-ACAGGTACGTTAATAGTTAATAGCGT-3', reverse primer 5'-ATATTGCAGCAGTACGCACACA-3', probe 5'- ACACTAGCCATCCTTACTGCGCTTCG-BBQ-3'^35^). Blood samples were taken at baseline, day 7 and day 28 visits. All 26 PCR-positive contacts seroconverted by day 28. Of the 28 PCR-negative contacts, one seroconverted and was subsequently excluded (Fig. 3a).

### Epitope prediction and informatics

Consensus sequences were generated from available sequences for SARS-CoV-2; huCoV-OC43; huCoV-HKU1 obtained from NCBI prior to April 27th 2020. Sequence alignments were conducted in Geneious Prime (version 2020.1.2) using Clustal Omega (Version 1.2) with default parameters. Potential cross-reactive epitopes were predicted based on the integration of SYFPEITHI and the Immune Epitope Database (IEDB) using shared sequences (longer than 9-mer) between SARS-CoV-2 and the two beta-huCoVs. The score given by SYFPEITHI is based on the frequency of amino acids (aa) that occur in anchor positions. A cut-off (score ≥ 20) was applied in SYFPEITHI and the results in IEDB were selected for a binding affinity (IC50) threshold of 500 nM by using NetMHCpan 4.0 with default parameters. The consensus sequences for each protein in the three coronaviruses were uploaded into IEDB. 9–11-mer peptides with IC50 < 50 (NetMHCpan 4.0 with default parameters) were recognised as strong binding peptides for MHC class I molecules. For MHC class II molecules, only 15-mer peptides were analysed and a cut-off IC50 < 50 (NetMHCpan 3.2 with default parameters) was applied.

### Dual cytokine FLISpot

Cryopreserved PBMCs were rested overnight at 37 °C and 5% CO_2_ at high density (1.5 × 10^7^/ml) before stimulation in a dual-cytokine FLISpot. All time points from an individual were thawed for stimulation and FLISpot analysis in the same run. Stimuli used included protein-spanning peptide pools (15 mers with 11 aa overlap, >70% purity) specific for spike (S), nucleocapsid (N), membrane (M) and envelope (E) proteins (Catalogue numbers PM-WCPV-S-2, PM-WCPV-NCAP-2, PM-WCPV-NCAP-2, PM-WCPV-VME-2 and PM-WCPV-VEMP-2 respectively from JPT, Berlin, Germany) of SARS-CoV-2. Individual peptides that represent the cross-reactive pool were custom synthesised by Genscript. PBMCs were stimulated with peptide pools (1 µg/peptide/ml), positive control pool of CMV, EBV and influenza epitopes (CEF) (1 µg/peptide/ml) (Catalogue no. 3616-1, Mabtech AB, Stockholm, Sweden) or soluble anti-CD3 and anti-CD28 (Catalogue no. FSP-0102-10, Mabtech AB, Stockholm, Sweden, used 1 in 1000 as per manufacturer’s instructions) and incubated on pre-coated IFN-γ and IL-2 capture plates (Catalogue no. FSP-0102-10, Mabtech AB, Stockholm, Sweden) for 20 h. Plates were processed as per manufacturer’s instructions, using anti-IFN-γ detector at 1 in 200 and anti-IL-2 detector at 1 in 500). The plates were read, and spots enumerated on an AID iSpot (AID, Strassberg, Germany) and results expressed as DMSO negative control subtracted spot forming cells (SFC) per 1 × 10^6^ PBMCs.

### Generation and restimulation of short-term antigen-specific T cell lines

To confirm specificity and proliferative capacity of memory T cell responses, we generated short term antigen-specific T cell lines: 500,000 PBMCs were stimulated with 2.5 µg/peptide/ml CEF or cross-reactive pool and 10 ng/ml IL-2. Medium was refreshed with RPMI + 10% FCS + 10 ng/ml IL-2 on day 3 and 6, and RPMI + 10% FCS alone on day 9. On day 12, lines were harvested, counted and plated at 5 × 10^4^ per well, restimulated (1 µg/peptide/ml or anti-CD3/anti-CD28 as per manufacturer’s instructions) and incubated on pre-coated IFN-γ capture ELISpot plates (Catalogue number 3420-2HPW-10, Mabtech AB, Stockholm, Sweden) for 20 h. Plates were developed as per manufacturer’s instructions, using anti-IFN-γ detection antibody diluted 1 in 200).

### Hybrid double antigen binding assay (DABA) for total Anti-RBD

Total antibodies specific for SARS-CoV-2 RBD were quantified by a highly sensitive and specific double antigen binding assay, as described previously^36^. Briefly, solid phase 96-microwells plates were coated with 100 μl of S1 antigen at a concentration of 5 μg/ml. Control and test sera were added to washed plates and incubated for 1 h at 37degrees. Detection was performed with RBD-HRP and development with TMB and plates read spectrometrically at 450–630 nm. The cut-off was established by adding 0.1 to the average of optical density (OD) obtained for three negative controls assayed in each run. The signal/cut-off value (binding ratio, BR) for each sample was determined by dividing the sample OD by the cut-off OD. A sample was considered positive if BR ≥ 1. United Kingdom Patent Application No. 2011047.4 for “SARS-CoV-2 antibody detection assay” has been filed.

### Exposure score

An approximate exposure score was computed based on the relationship with and exposure to the index case as per the criteria outlined in Supplementary Table 3.

### Human endemic coronavirus antibodies

Baseline serum samples were assayed for the presence of antibodies specific to Nucleoprotein of OC43, HKU1, 229E and NL-63 human endemic coronaviruses by Recomline (Mikrogen, Munich, Germany) as per manufacturer’s instructions. Briefly, serum samples were diluted 1 in 100 and incubated with test strips for 1 h. Detection antibody was used 1 in 100 (Rabbit anti-human IgG.)

### Statistical analysis

Data storage and curation was in Excel for Microsoft Office 365 (16.0.13801.21004) 32-bit. Graphs were generated and statistical analyses were performed in Prism 9. Binary logistic regression was performed in R (Version 4.0.3(2020-10-10) - “Bunny-Wunnies Freak Out”) with IL-2-SFC against the cross-reactive pool as a predictive variable and PCR positivity as the outcome (positive = 1 and negative = 0).

### Reporting summary

Further information on research design is available in the Nature Research Reporting Summary linked to this article.

## Supplementary information

Supplementary Information

Description of additional Supplementary File

Supplementary Data 1

Reporting Summary

## Data Availability

The datasets generated are available in the Supplementary Information file, or will be made available from the authors upon reasonable request. The identifiers of the sequences used in the bioinformatic analyses are detailed in the Supplementary Data 1. Source data are provided with this paper.

## Source data

Source Data
