# Supplementary material for: Cross-reactive memory T cells associate with protection against SARS-CoV-2 infection in COVID-19 contacts
Source: Nat Commun. 2022 Jan 10;13:80. doi: 10.1038/s41467-021-27674-x (PMC8748880; doi:10.1038/s41467-021-27674-x)
Supplement: Supplementary file 1 — Supplementary Information [file 41467_2021_27674_MOESM1_ESM.pdf]

Supplementary Information

"Cross-reactive memory T cells associate with protection against SARS-CoV-2 infection in COVID-19 contacts."

Kundu et al.,

Contact Info

Corresponding author, Dr Rhia Kundu.

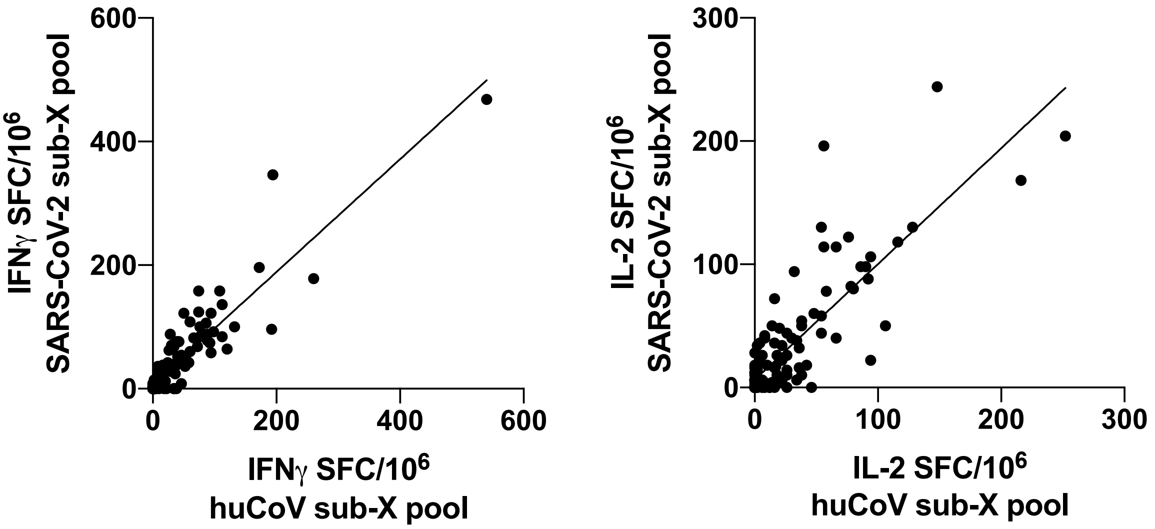

Supplementary Figure 1: Equivalent recognition of SARS-CoV-2 and huCoV-sequence derived cross-reactive peptides in T cells induced by SARS-CoV-2 infection.

PBMCs samples from indexes (n=14) and their PCR positive contacts (n=23) at the baseline (n=37), D7 (n=31), D14 (n=11) and D28 (n=27) time points were assayed by fluorospot for IFN-γ and IL-2 secreting T cells that recognise both SARS-CoV-2 and huCoV-sequence derived cross-reactive epitopes (n=107 total data points). Data are DMSO-negative control subtracted and presented as SFC per million PBMC.

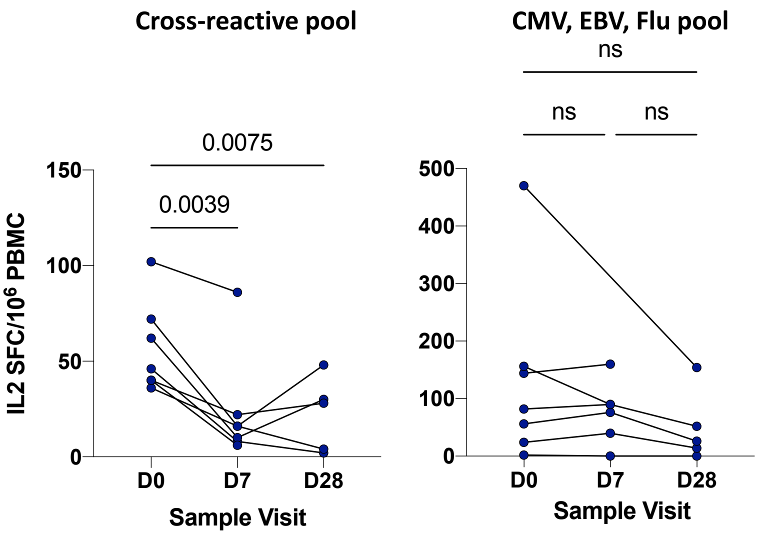

Supplementary Figure 2: Dynamics of IL-2 secreting cross-reactive T cells during follow up.

PBMCs sampled from COVID-19 contacts at the baseline, D7 and D28 visit were rested overnight at high density prior to stimulation with 1µg/ml cross-reactive peptide pool cultured for 20 hours in a FLISpot assay to detect IL-2-and IFN-γ secreting T cells. IL-2 SFC across the visits is plotted for PCR-negative individuals with >22 SFC/10^6 PBMCs at the baseline visit. Individual fluctuations in frequency are displayed for n=7 PCR negative contacts that had a baseline cross-reactive T cell response >22 SFC/million PBMC. Adjusted p-values from Tukey's multiple comparisons test are shown.

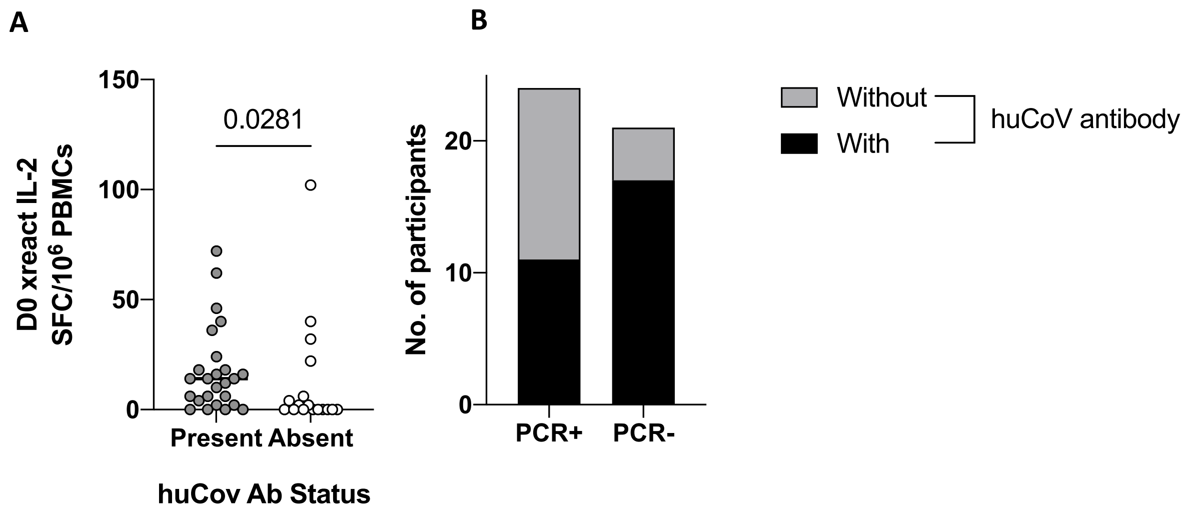

Supplementary Figure 3: Presence of antibodies against NL-63, HKU1, OC43 or 229E N protein are associated higher frequencies of cross-reactive T cells and more prevalent in PCR-negative contacts.

Baseline serum samples were assayed for antibodies specific to N-protein of hu-CoVs NL-63, HKU1, OC43 and 229E. The frequency of baseline cross-reactive IL-2 secreting T cells is plotted for all contacts with or without huCoV antibodies (A). P-values are from a two-sided Mann-Whitney U; n=25 individuals with huCoV-N antibodies, and n=17 who did not. The proportions of PCR-positive and PCR-negative contacts with huCoV antibodies are shown in (B).

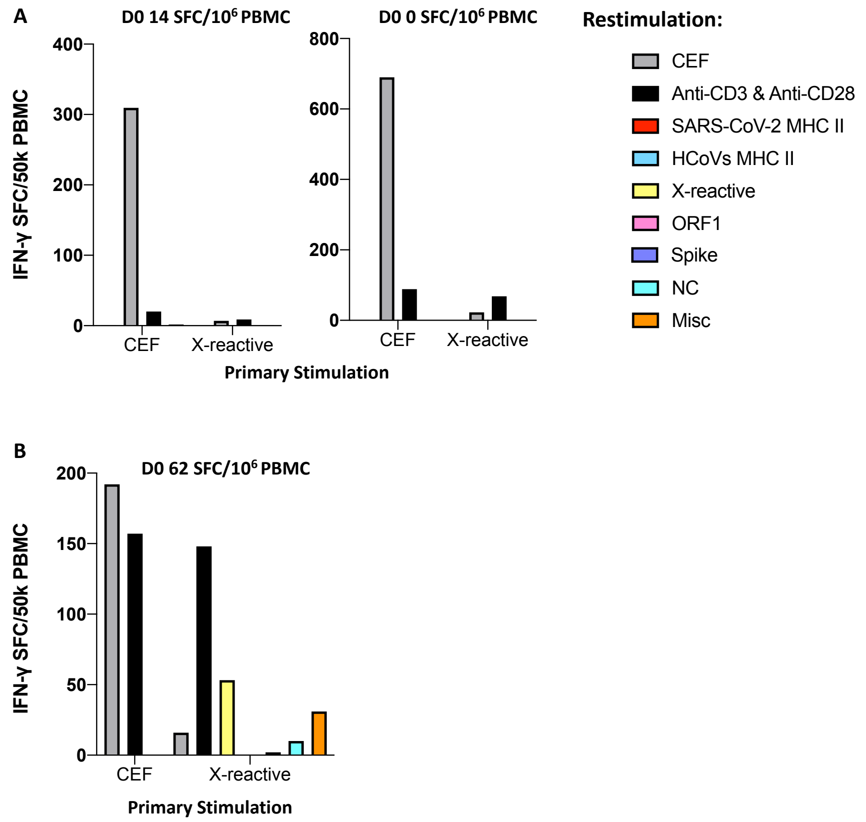

Supplementary Figure 4: Specificity of Short-Term T Cell lines from Low and High Frequency Responders to the Cross-Reactive Pool. Supplementary Figure 3: Specificity of Short-Term T Cell Lines from Low and High Frequency Responders to the Cross-Reactive Pool.

Baseline visit PBMCs ($5x10^5$ at $2x10^6$/ml) from 2 individuals with low (A) and one individual with high (B) frequencies of cross-reactive IL-2-secreting T cells were stimulated with 2.5ug/peptide/ml CEF or cross-reactive pool and 10ng/ml IL-2 in RPMI+10%FCS. Media was refreshed with RPMI + 10% FCS + 10ng/ml IL-2 on day 3 and 6, and RPMI +10% FCS alone on day 9. On day 12, lines were harvested, counted and plated at $5x10^4$ per well and restimulated 1ug/peptide/ml and incubated on pre-coated IFN-γ capture ELISpot plates for 20 hours. Plates were developed as per manufacturer's instructions. Each graph represents data from one experiment per individual performed in duplicate.

Supplementary Table 1: Peptides included in the cross-reactive pool

Sequence	Target	MHC-restriction	HLA-alleles	Study
QYIKWPWYIW	Spike	MHC-I	HLA-A*24:02	
EAEVQIDRLI	Spike	MHC-I	HLA-B*49:01	
KLIANQFNSA	Spike	MHC-I	HLA-A*02:03	
RLITGRLQSL	Spike	MHC-I	HLA-A*02:03	
RSFIEDLLF	Spike	MHC-I	HLA-B*58:01	
SFIEDLLFNKV	Spike	MHC-I	HLA-A*02:01; HLA-A*02:06	
SVLNDILSRL	Spike	MHC-I	HLA-A*02:03	
VQIDRLITGR	Spike	MHC-I	HLA-A*68:01	
VVNQNAQAL	Spike	MHC-I	HLA-A*26	
NQKLIANQFNSAIGK	Spike	MHC-II	HLA-DRB1*13:02	
QKFNGLTVLPPLLTD	Spike	MHC-II	HLA-DRB1*01:01	
SSNFGAISSVLNDIL	Spike	MHC-II	HLA-DRB1*01:01	
KRSFIEDLLFNKVTL	Spike	MHC-II	HLA-DPA1*01:03; HLA-DPB1*02:01	
TTDPSFLGRY	Orf1	MHC-I	HLA-A*01	Nedle et al.,
KLFAAETLK	Orf1	MHC-I	HLA-A*03	Nedle et al.,
TPKYKFVRI	Orf1	MHC-I	HLA-B*08	Nedle et al.,
DLKGKYVQI	Orf1	MHC-I	HLA-B*08	Nedle et al.,
IEYPIIGDEL	Orf1	MHC-I	HLA-B*40	Nedle et al.,
LDDFVEIIKSQDLSV	Orf1	MHC-II	HLA-DRB1*11	Nedle et al.,
WVLNNDYYR	Orf1	MHC-I	HLA-A*68:01; HLA-A*31:01; HLA-A*33:01	
YRLANECAQV	Orf1	MHC-I	HLA-A*02:03; HLA-A*02:01; HLA-A*02:06	
FVDGVPFVV	Orf1	MHC-I	HLA-A*02:06; HLA-A*02:01	
HEFCSQHTM	Orf1	MHC-I	HLA-B*40:01	
FVSLAIDAY	Orf1	MHC-I	HLA-B*35:01	
VLYYQNNVF	Orf1	MHC-I	HLA-B*15:01	
SVFNICQAV	Orf1	MHC-I	HLA-A*68:02; HLA-A*02:06; HLA-A*02:03	
RILGAGCFV	Orf1	MHC-I	HLA-A*02:06	
TQMNLKYAI	Orf1	MHC-I	HLA-A*02:06	
NVNRFNVAI	Orf1	MHC-I	HLA-A*68:02	
SLAIDAYPL	Orf1	MHC-I	HLA-A*02:01	
AAVDALCEK	Orf1	MHC-I	HLA-A*11:01	

KDGIIWVATEGALNT	NC	MHC-II	HLA-DRB1*01, -DRB1*04, -DRB1*11	Nedle et al.,
GTWLTYTGAIKLDDK	NC	MHC-II	HLA-DRB1*01, DRB1*07, DRB1*15	Nedle et al.,
RWYFYYLGTGPEAGL	NC	MHC-II	HLA-DRB1*04	Nedle et al.,
ASWFTALTQHGKEDL	NC	MHC-II	HLA-DRB1*04, DRB1*11	Nedle et al.,
ASAFFGMSRIGMEVT	NC	MHC-II	HLA-DRB1*01, DRB1*04, DRB1*07, DRB1*11	Nedle et al.,
LLLLDRLNQLESKMS	NC	MHC-II	HLA-DRB1*04, DRB1*15	Nedle et al.,
KPRQKRTA	NC	MHC-I	HLA-B*08	
PRWYFYYLGT	NC	MHC-I	HLA-B*08:01; HLA-B*14:02	
RWYFYYLGTGPEAGL	NC	MHC-II	HLA-DRB1*01:01	
RTFKVSIWNLDY	ORF6	MHC-I	HLA-A*01:01	Nedle et al.,
YEGNSPFHPL	ORF7	MHC-I	HLA-B*40	Nedle et al.,
FYVYSRVKNLNSSRV	ORF 4 Envelope	MHC-II	HLA-DRB1*04, DRB1*11	Nedle et al.,
IWNLDYIINLIIKNL	ORF6	MHC-II	HLA-DRB1*04, DRB1*07, DRB1**15	Nedle et al.,
QEEVQELYSPIFLIV	ORF7	MHC-II	HLA-DRB1*01, -DRB1*07	Nedle et al.,
SKWYIRVGARKSAPL	ORF8	MHC-II	HLA-DRB1*01, DRB1*11	Nedle et al.,

Supplementary Table 2: SARS-CoV-2 and huCoV peptides comprising the sub-X pools.

	SARS-CoV-2 Pool	huCoV-Pool	
Peptide	SARS-COV-2	OC43	HKU1
ii_S1	NQKLIANQFNSAIGK	QKLIANAFNNALHAI	-
ii_S2	QKFNGLTVLPPLLTD	-	VQSFNGIKVLPPILS
ii_S3	SSNFGAISSVLNDIL	-	LFNKFGAISSSLQEI
ii_S4	KRSFIEDLLFNKVTL	-	SRSFFEDLLFDKVKL
ii_N1	RWYFYYLGTGPEAGL	PRWYFYYLGTGPHAK	-

Supplementary Table 3: A priori defined scoring system for relationship with the index.

Relationship with Index	Score
Partner/Parent to ≤16 child/≤16 child/Sibling with shared bedroom	100
Parent to child >16/>16 child/Sibling without shared bedroom but same house	80
Housemate/Residential employee	60
Non-Residential work colleague/employee	40
Non-Residential friend/Non-Residential relative	20
Unknown non-household	10
