# Supplementary material for: Cross-reactive memory T cells associate with protection against SARS-CoV-2 infection in COVID-19 contacts
Source: Nat Commun. 2022 Jan 10;13:80. doi: 10.1038/s41467-021-27674-x (PMC8748880; doi:10.1038/s41467-021-27674-x)
Supplement: Supplementary file 2 — Description of additional Supplementary File [file 41467_2021_27674_MOESM2_ESM.pdf]

**Description of additional data supplementary data file**

File Name: Supplementary Data 1
Description: A table of accession IDs used in the manuscript analysis.
