# Supplementary material for: Cross-reactive memory T cells associate with protection against SARS-CoV-2 infection in COVID-19 contacts
Source: Nat Commun. 2022 Jan 10;13:80. doi: 10.1038/s41467-021-27674-x (PMC8748880; doi:10.1038/s41467-021-27674-x)
Supplement: Supplementary file 4 — Reporting Summary [file 41467_2021_27674_MOESM4_ESM.pdf]

Corresponding author(s): Dr Rhia Kundu
Last updated by author(s): Nov 10, 2021

Reporting Summary

Nature Portfolio wishes to improve the reproducibility of the work that we publish. This form provides structure for consistency and transparency in reporting. For further information on Nature Portfolio policies, see our Editorial Policies and the Editorial Policy Checklist.

Statistics

For all statistical analyses, confirm that the following items are present in the figure legend, table legend, main text, or Methods section.

n/a	Confirmed	
☐	☒	The exact sample size (n) for each experimental group/condition, given as a discrete number and unit of measurement
☐	☒	A statement on whether measurements were taken from distinct samples or whether the same sample was measured repeatedly
☐	☒	The statistical test(s) used AND whether they are one- or two-sided<br>*Only common tests should be described solely by name; describe more complex techniques in the Methods section.*
☐	☒	A description of all covariates tested
☐	☒	A description of any assumptions or corrections, such as tests of normality and adjustment for multiple comparisons
☐	☒	A full description of the statistical parameters including central tendency (e.g. means) or other basic estimates (e.g. regression coefficient) AND variation (e.g. standard deviation) or associated estimates of uncertainty (e.g. confidence intervals)
☐	☒	For null hypothesis testing, the test statistic (e.g. F, t, r) with confidence intervals, effect sizes, degrees of freedom and P value noted<br>*Give P values as exact values whenever suitable.*
☒	☐	For Bayesian analysis, information on the choice of priors and Markov chain Monte Carlo settings
☒	☐	For hierarchical and complex designs, identification of the appropriate level for tests and full reporting of outcomes
☒	☐	Estimates of effect sizes (e.g. Cohen's d, Pearson's r), indicating how they were calculated

Our web collection on statistics for biologists contains articles on many of the points above.

Software and code

Policy information about availability of computer code

Data collection	No software was used for data collection, but storage was in Excel for Microsoft Office 365 (16.0.13801.21004) 32-bit
Data analysis	Binary logistic regression was preformed in R (Version 4.0.3(2020-10-10) - "Bunny-Wunnies Freak Out"; Copyright(C) 2020. The R fondation for statistical computing; Platform x86_64-apple-darwin17.0 (64-bit) using the glm function, through R Studio 1.3.1093.<br><br>Sequence alignments were conducted in Geneious Prime (version 2020.1.2) using Clustal Omega (Version 1.2) default parameters.<br><br>Figure and plots were generated in Graph Pad Prism (version 9).<br><br>Data storage and curation was in Excel for Microsoft Office 365 (16.0.13801.21004) 32-bit

For manuscripts utilizing custom algorithms or software that are central to the research but not yet described in published literature, software must be made available to editors and reviewers. We strongly encourage code deposition in a community repository (e.g. GitHub). See the Nature Portfolio guidelines for submitting code & software for further information.

Data

Policy information about availability of data

All manuscripts must include a data availability statement. This statement should provide the following information, where applicable:

- Accession codes, unique identifiers, or web links for publicly available datasets
- A description of any restrictions on data availability
- For clinical datasets or third party data, please ensure that the statement adheres to our policy

We have included the statement: "The datasets generated are available in the Source Data file. The identifiers of the sequences used in the bioinformatic analyses are detailed in the Supplementary Table 3." in the Methods

Field-specific reporting

Please select the one below that is the best fit for your research. If you are not sure, read the appropriate sections before making your selection.

☒ Life sciences ☐ Behavioural & social sciences ☐ Ecological, evolutionary & environmental sciences

For a reference copy of the document with all sections, see nature.com/documents/nr-reporting-summary-flat.pdf

Life sciences study design

All studies must disclose on these points even when the disclosure is negative.

Sample size	Sample size was dictated by the logistics of recruiting acutely exposed (1-5 days post symptom onset in the index) individuals within an unfolding pandemic, during the first and second wave of SARS-CoV-2 in the UK (to ensure a truly naive cohort). To enter a household, our nursing teams required full PPE and adherence to stringent infection control measures. As such each participant recruited represents a considerable number of person-hours to ensure the safe generation of a globally unique set of samples from previously unexposed individuals so early on in their primary SARS-CoV-2 exposure.<br>With n=26 PCR positive and n=26 PCR negative individuals, an effect size calculation indicated that we would have 80% power to detect an effect size equivalent to a 2 fold difference in the means of cross-reactive T cell responses at baseline between the two groups (as calculated in R using the pwr package). The effect size we have observed is equivalent to a 3-fold difference in mean frequency of cross-reactive T cells between the two groups.
Data exclusions	Our analyses were restricted to COVID-19 contacts who were seronegative at baseline and where household transmission has occurred (ie for every PCR negative contact, there is at least one PCR positive contact of the same index). Individuals who were PCR negative but seroconverted during follow up were excluded as having an unclear outcome (n=1). These are described in the manuscript.
Replication	Direct replication of fluorospot T cell assays on the PBMC samples was no possible due to restricted cell numbers. However, fluorospot analyses were performed in duplicate in a demonstrably reproducible assay (CVs consistently <10%) which includes a countable positive control (25,000 PBMC are stimulated with anti-CD3 and anti-CD28 to activate T cells); this acts as a calibrator for the accuracy of counting out the cells and sample quality across the different time points for the assay, supporting subsequent interpretation of the data. Control samples with known frequencies of antigen-specific T cells were also run alongside the test samples to ensure the validity of the assay run. DABA assay reproducibility data is cited in the manuscript (Tedder, R. S. et al. Detection and Quantification of Antibody to SARS- CoV-2 Receptor Binding Domain Provides Enhanced Sensitivity, Specificity and Utility. SSRN Electron. J. (2021) doi:10.2139/ssrn.3739821.). GIven the limited amount of serum and volume demands from other workstreams, the DABA was not repeated within this cohort.
Randomization	Randomization did not occur for this prospective observational cohort study. All potential participants in the recruitment area whose index case had a symptom onset within 3 days who could be contacted with the available nursing staff were contacted for potential participation. In addition to the demographic skew associated with the geographical issues with sampling individuals, the nature of the household visits mean there is likely a significant degree of self-selection in those who would be willing to participate.
Blinding	Nurses collecting and storing participant information were blinded to infection outcome during the initial visit, given the prospective nature of the sampling. Technicians running PCR, antibody and T cell fluorospot assays were blinded to results from the other assays and the symptomatic status of the participants, as well as all demographic factors. Data analysis could not be blinded due to the limited available of individuals to perform the work and the need to apply exclusion criteria.

Reporting for specific materials, systems and methods

We require information from authors about some types of materials, experimental systems and methods used in many studies. Here, indicate whether each material, system or method listed is relevant to your study. If you are not sure if a list item applies to your research, read the appropriate section before selecting a response.

Materials & experimental systems

n/a	Involved in the study
☐	☒ Antibodies
☒	☐ Eukaryotic cell lines
☒	☐ Palaeontology and archaeology
☒	☐ Animals and other organisms
☐	☒ Human research participants
☒	☐ Clinical data
☒	☐ Dual use research of concern

Methods

n/a	Involved in the study
☒	☐ ChIP-seq
☒	☐ Flow cytometry
☒	☐ MRI-based neuroimaging

Antibodies

Antibodies used

All antibodies used were within validated kits from Mabtech (FSP-0102-10)

Validation

The mabtech fluorospot assays are a commercial kit and therefore subject to intense validation(https://www.mabtech.com/sites/default/files/datasheets/FSP-0102-2-10.pdf). We have performed our own validation in house for the purpose of TB and common positive control antigen (Casey et al 2018)

Human research participants

Policy information about studies involving human research participants

Population characteristics

Demographic data from the cohort are detailed in Table 1 and Source data files.

Recruitment

Household contacts of confirmed COVID-19 index cases were recruited through the national test and trace system with Public Health England, in the United Kingdom. Mobile nursing teams prioritized households close to the study teams location at Paddington, North West London. As such, there is a skew towards white, affluent participant's, a point which we draw attention to in the manuscript itself. Our nurses actively tried to increase the diversity of recruitment by prioritizing BAME staff to contact BAME potential participants, in case that would make individuals feel more comfortable about participation, which involved a team entering their home for invasive sampling and questioning for case report forms. As such, there will also be a large degree of self-selection in our study population towards affluent individuals with the financial means to take time out to participate, and also allow nursing teams into their own home, and the biological implications from this. Our group actively engages in PPIE activities in vulnerable and neglected groups for the long term improvement of diversifying participation in medical research within the UK.

Ethics oversight

North West- Greater Manchester East Research Ethics Committee, REC reference 20/NW/0321, IRAS ID 282820. We have referenced this in the manuscript.

Note that full information on the approval of the study protocol must also be provided in the manuscript.
